# Mitoxantrone resistance in a small cell lung cancer cell line is associated with ABCA2 upregulation

**DOI:** 10.1038/sj.bjc.6601863

**Published:** 2004-05-18

**Authors:** R Boonstra, H Timmer-Bosscha, J van Echten-Arends, D M van der Kolk, A van den Berg, B de Jong, K D Tew, S Poppema, E G E de Vries

**Affiliations:** 1 Department of Pathology and Laboratory Medicine, University Hospital Groningen, The Netherlands; 2 Department of Medical Oncology, University Hospital Groningen, The Netherlands; 3 Department of Genetics, University Hospital Groningen, The Netherlands; 4 Department of Pharmacology, Fox Chase Cancer Center, Philidelphia, Pennsylvania 19111, USA

**Keywords:** CGH, multidrug resistance, mitoxantrone, GLC4-MITO, ABCA2

## Abstract

The aim of this study was to find factors that could explain the accumulation difference of mitoxantrone in the BCRP1-negative GLC4-MITO cell line compared to GLC4. Comparative genomic hybridisation (CGH) was applied to determine chromosomal differences between GLC4 and GLC4-MITO. Comparative genomic hybridisation analysis revealed gain of 2q, 6p, 9q, 13q, 14q, 15q, 19q and Xp and loss of 1p, 2q, 3p, 3q, 4q, 6q, 8q, 11p, 16p, 17q, 18p, 20p and Xq. In the over-represented chromosomal areas, seven transporter genes were identified: ABCB6, ABCB2 (TAP1), ABCB3 (TAP2), ABCF1 (ABC50), ABCC10 (MRP7), ABCA2 (ABC2) and ABCC4 (MRP4). No RNA or protein upregulation was observed for ABCB6, ABCF1, ABCC10, ABCC4, ABCB2 and ABCB3, but an increased expression was detected for ABCA2 mRNA in GLC4-MITO. ABCA2 is known to be involved in resistance to estramustine. In the MTT assay, GLC4-MITO was two-fold resistant to estramustine compared to GLC4. Coincubation with estramustine and mitoxantrone increased mitoxantrone accumulation in GLC4-MITO, while this was not affected in GLC4. This suggests that estramustine is able to block mitoxantrone efflux in GLC4-MITO cells. These data reveal that cellular reduction of mitoxantrone in a mitoxantrone-resistant cell line is associated with overexpression of ABCA2.

The treatment of patients with anticancer agents is still seriously hampered by the occurrence of resistance against standard anticancer drugs and crossresistance to many other cytotoxic agents. The ATP-binding cassette transporter (ABC) superfamily has been implicated as a major contributor to the multidrug-resistance phenotype (Klein *et al*, 1999). The most studied members are the P-glycoprotein (P-gp) encoded by the MDR1 gene (Ambudkar *et al*, 1999) and the MDR-related protein (MRP) family of the ABC protein transporters, especially the MRP1 gene (Borst *et al*, 1999). Currently, 48 human ABC transporters are known (Müller M (2001). *Transporters in the liver and ATPbinding Casette (ABC)-Proteins October 2002 Internet communication*, http://nutrigene.4t.com/humana bc.htm). In addition, it has become clear that often more than one mechanism is involved in multidrug-resistance of tumor cells.

Resistance to the anticancer drug mitoxantrone has been associated with several mechanisms including drug accumulation defects and reduction in its target proteins topoisomerase II*α* and *β* (Withoff *et al*, 1996). Recently, overexpression of the breast cancer-resistance half-transporter protein (BCRP1) was found to be responsible for the occurrence of mitoxantrone resistance in a number of cell lines (Doyle *et al*, 1998; Miyake *et al*, 1999; Ross *et al*, 1999; Litman *et al*, 2000). However, not all mitoxantrone-resistant cell lines express BCRP1 (Hazlehurst *et al*, 1999; Nielsen *et al*, 2000). The efflux pump responsible for the mitoxantrone resistance in these cell lines is as yet unknown. The mitoxantrone-resistant GLC4 subline GLC4-MITO exhibits a reduced mitoxantrone accumulation (Withoff *et al*, 1996). Scheffer *et al* (2000) had already shown that there was no BCRP1 protein expression in GLC4-MITO using BXP-34 antibody . This suggests that a different efflux pump may be involved in mitoxantrone resistance in GLC4-MITO.

We used comparative genomic hybridisation (CGH) to determine chromosomal differences between the human small cell lung cancer cell line (SCLC) GLC4 and its mitoxantrone-resistant subline. Comparison of the cell lines showed gains of specific chromosomal regions. Screening of several databases revealed seven known efflux pumps located in the amplified regions. A possible involvement of these pumps was analysed by reverse transcriptase–polymerase chain reaction (RT–PCR) for mRNA expression, cytotoxicity with the microculture tetrazolium assay (MTA), drug accumulation by flow cytometry analysis and immunocytochemistry for protein expression.

## MATERIAL AND METHOD

### Cell lines

For this study, we used two cell lines: GLC4, a drug-sensitive human SCLC cell line and GLC4-MITO, a GLC4 subline with an *in vitro* induced mitoxantrone resistance (Zijlstra *et al*, 1986; Withoff *et al*, 1996). GLC4-MITO was previously shown to exhibit a 60% reduced topoisomerase II*α* compared to GLC4 and no topoisomerase II*β* RNA expression at all. In addition, a decreased mitoxantrone accumulation was found. There was a 3.6-fold crossresistance to doxorubicin without reduction in doxorubicin accumulation (Withoff *et al*, 1996). Earlier studies detected no overexpression or activation of P-gp, MRP1 (multidrug-resistant protein) and LRP (lung-resistance protein) in the GLC4-MITO cell line (Withoff *et al*, 1996). Both cell lines were cultured in RPMI 1640 supplemented with 10% heat-inactivated fetal calf serum, (37°C, 5% CO_2_). GLC4-MITO was incubated twice monthly with 285 nM mitoxantrone to maintain the mitoxantrone resistance. Before performing the experiments described below, cells were cultured drug free for 14 days. Routinely mitoxantrone sensitivity was checked by MTA every 3 months. A mean mitoxantrone-resistance factor of 33-fold was established. For the RT–PCR experiments, we used MCF7, a human breast carcinoma cell line and MCF7-MX, its 1395-fold mitoxantrone-resistant subline with increased BCRP1 mRNA expression compared to MCF7 (Ross *et al*, 1999; Volk *et al*, 2000) as controls. For the MTA experiments with estramustine, we included GLC4-ADR, a 150-fold doxorubicin-resistant GLC4 subline with increased MRP1 and LRP expression but no reduced mitoxantrone accumulation (Zijlstra *et al*, 1987) as a control.

### Cytogenetics

The cell cultures were harvested and chromosome preparations were made using standard cytogenetic techniques. The chromosomes were G-banded using 0.1% pancreatin (Sigma, Zwijndrecht, the Netherlands) and 10% Giemsa. A total of 10 metaphases from the GLC4 and the GLC4-MITO cell line were analysed. Karyotypes were described according to the ISCN (1995). chromosomal pattern of GLC4-MITO was compared with that of GLC4.

### Comparative genomic hybridisation

Cells of subconfluent cultures were harvested and genomic DNA was extracted according to standard methods. Comparative genomic hybridisation was performed as described by Kallioniemi *et al* (1992) with some adjustments. Approximately 1 *μ*g of test DNA and 1 *μ*g of reference DNA were labelled by nick translation with either Biotin-16-dUTP or digoxigenin-11-dUTP (Roche Molecular Biochemicals, Almere, Netherlands). Aliquots of 400 ng labelled test DNA and control DNA were ethanol precipitated with 50 *μ*g unlabelled human Cot1 DNA (Life Technologies, Breda, the Netherlands) and 10 *μ*g of salmon sperm DNA (Sigma, Zwijndrecht, the Netherlands). The DNA solution was dissolved in 15 *μ*l hybridisation mixture (50% deionized formamide, 2 × SSC, 10% dextran sulphate pH 7.0) and applied to a normal male metaphase slide (Vysis, Downers Grove, IL, USA). The slide with the DNA hybridisation solution was denatured at 74°C for 3 min and hybridized at 37°C for 72 h. Posthybridisation washes with 4 × saline-sodium citrate (SSC), 3 × 0.1 × SSC at 60°C were performed before immunochemical detection. Immunochemical detection was performed using streptavidin-FITC (Roche Molecular Biochemicals) and anti-DIG-TRITC (Roche Molecular Biochemicals, Almere, Netherlands) in 4 × SSC, Tween-20 and 1% fat-free powder milk during 1 h to detect the biotin-labelled tumour DNA and digoxigenin-labelled normal DNA. The slides were mounted with antifading solution containing 4,6-diamidino-2-phenylindole (DAPI), used as a counter stain (Vectashield, Vector laboratories, Burlingame, CA, USA).

The grey-scale images of the three different fluorochromes were captured using a Leica DMRA fluorescence microscope equipped with DAPI, FITC and TRITC filters (Chroma, Brattleboro, VT, USA), CCD camera (Cohu 4912 CCD camera, San Diego, CA, USA) and the image-capturing program QFISH (Leica, Cambridge, UK). The three captured images were combined and pseudocolor was applied matching the original colours of the fluorochromes. The ratio between the FITC (tumour cell line) and TRITC (normal) fluorescence was calculated with use of the QCGH software program (Leica, Cambridge). For each case, the mean of the individual ratio profiles of 8–10 metaphase spreads was calculated. The GLC4 and the GLC4-MITO cell line were compared with normal DNA. In addition, we directly compared GLC4-MITO with its parent line GLC4.

### RNA extraction and RT–PCR

Total cellular RNA was isolated from 5 × 10^6^ cells using 1 ml of Trizol reagent (Life Technologies, Breda, the Netherlands). RNA was extracted, precipitated and washed according to the manufacturer's protocol. RNA (2 *μ*g) was reverse transcribed in 20 *μ*l of reverse transcriptase (RT) buffer (Life Technologies), supplemented with 1.8 mM dTTP (Promega Corp., Madison, WI, USA), 10 U of Moloney murine leukemia virus RT (Amersham-Pharmacia, Woerden, The Netherlands), 4.8 U RNAguard (Amersham-Pharmacia, Woerden, The Netherlands), 0.2 *μ*g pd(N)_6_ random primers (Amersham-Pharmacia,) and 3 mM dithiothreitol (Life Technologies). The reaction conditions were 65°C for 10 min and 37°C for 60 min. After this incubation, 30 *μ*l H_2_O was added up to a final volume of 50 *μ*l cDNA. Polymerase chain reaction analysis was performed in 25 *μ*l PCR buffer containing 2 *μ*l of cDNA using the primer pairs and conditions as described in Table 1

**Table 1:** Primer sequences and PCR conditions for the ATP-binding cassette transporter (ABC) genes that were located in the amplified chromosomal regions, BCRP1, MRP1, MRP2, MRP5 and the control genes hypoxanthine phosphoribosyltransferase (HPRT) and *β*-2 microglobulin, used for DNA amplification in the RT–PCR

**Gene**	**Sense primer (5′ → 3′)**	**Antisense primer (3′ → 5′)**	**Temperature (°C)**	**Cycles (*n*)**	**Product size (base pairs)**
ABCB6	GGG CCG TAT TGA GTT TGA GA	ATG TCC TGC CCA TCT ATT CG	95, 55, 72^a^	26	201
ABCC10 (MRP7)	CTC CCA CTG GAT CTC TCA GC	TCG CAT ACA CGG TGA GGT AG	95, 57, 72^a^	30	200
ABCF1 (ABC50)	GGA GTA CAC TGT GCG CTT CA	TCA GCA GCA GGA GTA GCG TA	95, 55, 72^a^	26	196
ABCA2 (ABC2)	GAG ATC CGC AGA GAG ATG GA	CTT CAG GAT GAG GTC CCA GA	95, 57, 72^a^	30	207
ABCC4 (MRP4)	AAT ACC CTT GGT TCC CCT TGG	ATC CTG GTG TGC ATC AAA CA	95, 55, 72^a^	32	202
ABCG2 (BCRP1)	CAG AGA TCA TAG AGC CTT CC	ACA CTC TGT AGT ATC CTC TG	95, 55, 72^a^	32	453
ABCC1(MRP1)	AAT GCG CCA AGA CTA GGA AG	ACC GGA GGA TGT TGA ACA AG	95, 56, 72^a^	29	990
ABCC2 (MRP2)	CTG GTT GAT GAA GGC TCT GT	CTG CCA TAA TGT CCA GGT TC	95, 58, 72^a^	31	1067
ABCC5 (MRP5)	GGA TAA CTT CTC AGT GGG	GGA ATG GCA ATG CTC TAA AG	95, 55, 72^a^	29	380
HPRT	CGT GGG GTC CTT TTC ACC AGC AAG	AAT TAT GGA CAG GAC TGA ACG TC	95, 55, 72^a^	27	380
*β*-2 microglobulin	CCA GCA GAG AAT GGA AAG TC	GAT GCT GCT TAC ATG TCT CG	95, 55, 72^a^	20	268

a For denaturation (30′′), annealing (30′′), extension (30′′).

.

The PCR reaction product bands were visualised by ethidium bromide staining. Densitometric scanning was performed with an Image Master VDS (Pharmacia, Woerden, The Netherlands), and optical density (OD) was expressed as OD × mm^2^ using the program Diversity One 1D (PDI, New York, NY, USA).

### Immunocytochemistry for ABCB2 (TAP1), ABCB3 (TAP2) proteins

Cytospin slides were prepared from cultured GLC4 and GLC4-MITO cells. The cytospins were fixed in acetone for 10 min, transferred to ice-cold methanol for 10 min and washed with phosphate-buffered saline (PBS), pH 7.4. The cytospins were incubated for 1 h at room temperature with the primary antibody ABCB2 (TAP1), ABCB3 (TAP2) (antibodies kindly provided by Dr J Neefjes, Dutch Cancer Institute, the Netherlands) diluted in 1% bovine serum albumin in PBS. Endogenous peroxidase was blocked with 0.3% H_2_O_2_ for 30 min. For ABCB3 and ABCB2 antibodies, the second step was performed with peroxidase-conjugated rabbit anti-mouse antibody (Dakopatts, Glosstrup, Denmark) supplemented with 1% human serum, followed by incubation with goat anti-rabbit antibody (Dakopatts). The visualisation was performed in a freshly prepared solution of 3-amino-9-ethylcarbazol (AEC), containing 0.03% H_2_O_2_ for 10 min. Counterstaining was performed using Mayer's haematoxylin. Control slides, in which PBS replaced the first antibody, were consistently negative.

### Cytotoxicity assay

Cytotoxicity of estramustine was determined using MTA as described before (Timmer-Bosscha *et al*, 1989). Before the assays were performed, cell growth studies were carried out, and the linear relationship of cell number to formazan crystal formation was checked. Each cell line was seeded at optimum density in order to test survival after at least two to three cell divisions had taken place in the control cells. For GLC4, 3 × 10^4^ cells ml^−1^ and for GLC4-ADR and GLC4-MITO, 7.5 10^4^ cells ml^−1^ were incubated continuously for 4 days with estramustine concentrations ranging from 1 to 100 *μ*M. Controls consisted of media without cells (background extinction) and cells incubated in microculture wells with medium without the drug. The surviving fraction was calculated by the ratio of mean extinction of test sample to mean extinction of untreated control sample. Inhibition concentration (IC)_50_, IC_70_ and IC_90_ were defined as the doses of estramustine inducing 50, 70, and 90% reduction in cell survival, respectively. Experiments were performed four times each in quadruplicate.

### Flow cytometric detection of mitoxantrone accumulation

The ability of tumour cell lines GCL4 and GLC4-MITO to extrude mitoxantrone in the absence or presence of the ABCA2 modulator estramustine, the P-gp inhibitor PSC833 (provided by Novartis Pharma Inc., Basel Switzerland), the BCRP1 inhibitor fumitremorgin C (FTC) (kindly provided by SE Bates) and the leukotriene D4 receptor antagonist and MRP inhibitor MK-571 (provided by Sanvertech, Heerhugowaard, The Netherlands) was measured in a fluorescence-activated cell sorting (FACS) assay (Van der Kolk *et al*, 2002). Cells (1 × 10^6^) were preincubated with these inhibitors for 30 min at 37°C, 5% CO_2_, in the following combinations: RPMI 1640 medium alone (0.5 ml), RPMI 1640 medium plus 10 *μ*M FTC, or plus 2 *μ*g ml^−1^ PSC833 or plus 20 *μ*M MK-571. Thereafter, 3 *μ*M mitoxantrone was added and the cells were incubated for 60 min at 37°C, 5% CO_2._

Cells (1 × 10^6^) were preincubated with and without 10 or 25 *μ*M estramustine (modulator of ABCA2), 3 *μ*M mitoxantrone was added and the cells were incubated for 60 min at 37°C, 5% CO_2_. Cells were washed with ice-cold RPMI 1640 medium. Fluorescence of mitoxantrone was analysed with a FACScalibur flow cytometer (Becton Dickson Medical Systems, Sharon, MA, USA), equipped with an argon laser. The viable cell population was gated by forward and sideways scatter characteristics. The mitoxantrone fluorescence of 10 000-gated events was logarithmically measured at a laser excitation wavelength of 635 nm through a 670 nm band-pass filter. Mitoxantrone accumulation was expressed as median fluorescence intensity (MFI). The effect of the various modulators was expressed as a shift of MFI of the mitoxantrone accumulation (Van der Kolk *et al*, 2002). Measurements were performed on duplicate samples and experiments were performed in triplicate.

### Statistics

For the statistical analysis of the data retrieved in the mitoxantrone accumulation and cytoxicity experiments, we used the paired or unpaired Student's *t*-test.

## RESULTS

Cytogenetics revealed a complex karyotype with marker chromosomes in both GLC4 and GLC4-MITO (data not shown). Comparison of the CGH results with cytogenetics showed that numerical differences detected with CGH were also observed in the karyotypes of GLC4 and GLC4-MITO.

**Figure 1:**
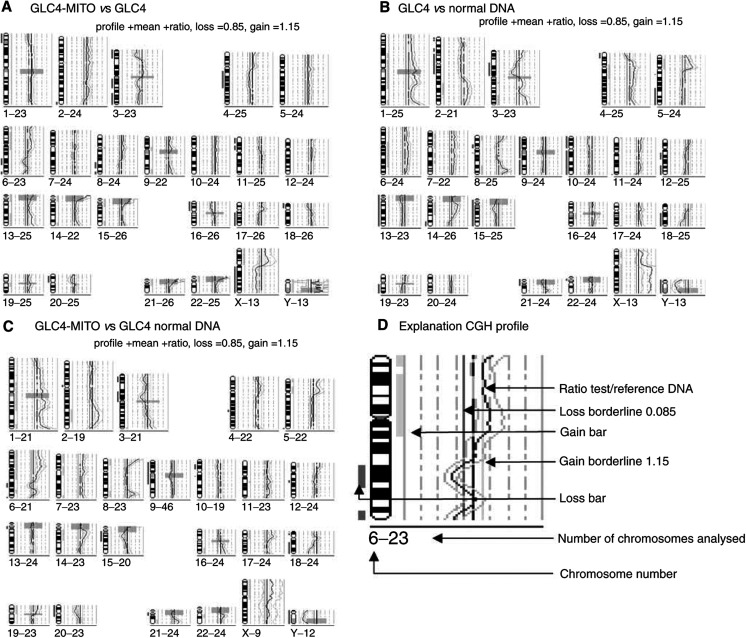
Comparative genomic hybridisation profiles of GLC4-MITO *vs* GLC4 and normal control DNA. (**A**) CGH profile of GLC4-MITO *vs* GLC4 (blue line) (**B**) CGH profile of GLC4 *vs* normal male DNA. (**C**) CGH profile of GLC4-MITO *vs* normal male DNA. (**D**) Explanation of schematic representation of a CGH profile gain >1.15(green bar) loss <0.85 (red bar).

Comparative genomic hybridisation was used to compare the mitoxantrone-resistant cell line with its sensitive parental line GLC4. In addition, we also compared both cell lines with a normal DNA sample (genomic DNA isolated from peripheral blood lymphocytes from a healthy person). Results of all CGH experiments are shown in Figure 1

. The CGH results of GLC4-MITO *vs* GLC4 are summarized in Table 2

**Table 2:** Copy number changes detected by CGH in GLC4-MITO *vs* GLC4

**Gain**
rev ish enh(2q34 6p25q14 9q34 13q21q34 14q32 15q24q26 19q10q13.1 Xp)
**Loss**
rev ish dim(1p36.3p34.1 2q37 3p36p22 3q11.2q23 3q25q29 4q13q28 6q22q24 6q26q27 8q22q23 8q24.2q24.3 11p15p14 11p12 16p 17q12q25 18p11 20p13p11.2 Xq)

CGH=comparative genomic hybridisation.

. Loss (17x) of chromosomal material was more frequent than gain (8x) in GLC4-MITO compared to GLC4. Relative to GLC4 no high level amplifications could be detected in GLC4-MITO using CGH. We detected loss of 3p (containing the topoisomerase II*β* gene) and 17q (containing the topoisomerase II*α* gene) in GLC4-MITO providing a plausible explanation for the previously identified reduction of topoisomerase activity (Withoff *et al*, 1996).

The genomic areas enriched in GCL4-MITO as compared with GLC4 were screened in a database for the presence of known drug resistance-related transporter genes (Müller M (2001) *Transporters in the Liver and ATPbinding Casette (ABC)-Proteins October 2002*. Internet communication, http://nutrigene.4t.com/humana bc.htm). We identified seven known transporter genes: ABCB6 (ABCB6) (Allikmets *et al*, 1996), ABCB2 (TAP1), ABCB3 (TAP2) (Bahram *et al*, 1991), ABCC10 (MRP7) (Hopper *et al*, 2001), ABCF1 (ABC50) (Richard *et al*, 1998), ABCA2 (ABC2) (Vulevic *et al*, 2001) and ABCC4 (MRP4) (Kool *et al*, 1997) (see Table 3

**Table 3:** Transporter genes located in gained chromosomal areas when GLC4-MITO was compared with GLC4 using CGH

**Name (symbol)**	**Chromosomal location**	**References**
ABCB6 (ABCB6)	2q33–q36	Allikmets *et al* (1996)
ABCB2 (TAP1)	6p21.3	Bahram *et al* (1991)
ABCB3 (TAP2)	6p21.3	Bahram *et al* (1991)
ABCC10 (MRP7)	6p21	Hopper *et al* (2001)
ABCF1 (ABC50)	6p21.33	Richard *et al* (1998)
ABCA2 (ABC2)	9q34	Vulevic *et al* (2001)
ABCC4 (MRP4)	13q32	Kool *et al* (1997)

).

**Figure 2:**
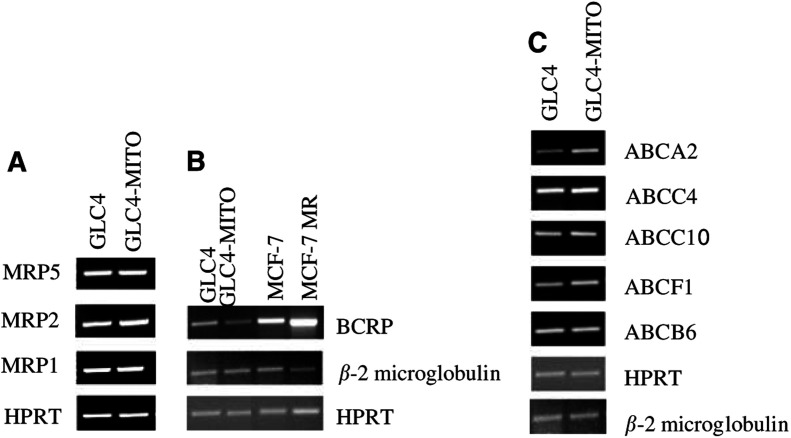
RT–PCR of selected efflux pump genes. (**A, B**): Expression of MRP1 MRP2 MRP5 BCRP *β* -2 microglobulin and HRTP mRNA in the mitoxantrone-resistant cell line GLC4-MITO and sensitive cell line GLC4. (**C**): Expression of ABCB6 ABCF1 ABCC10 ABCC4 ABCA2 *β* 2-microglobulin and HRTP in the mitoxantrone-resistant cell line GLC4-MITO and its sensitive parent line GLC4.

These transporter genes located at 2q, 6p, 9q and 13q, respectively, were analysed for overexpression at the RNA and/or protein level. ABCB2 and ABCB3 protein expression was screened by immunocytochemistry. Staining with the anti-ABCB2 antibody revealed a comparable, weakly positive, cytoplasmatic staining in GLC4 and GLC4-MITO. With the anti-ABCB3 antibody, a moderate to strong cytoplasmatic staining pattern was observed with no differences between both cell lines. Reverse transcriptase–polymerase chain reactions were performed for the other four transporter genes, for the BCRP1 gene (known to be involved in mitoxantrone resistance) and for MRP1, MRP2 and MRP5 as controls. Densitometric scanning of the PCR reaction product bands revealed similar expression levels for ABCB6, ABCF1, ABCC10, ABCC4, MRP1 and MRP5 in the parental cell line GLC4 as compared with resistant line GLC4-MITO. MRP2 was slightly higher in GLC4-MITO, but FACS results showed no difference in the effect of MRP inhibitor MK-571 on GLC4 and GLC4-MITO. Reverse transcriptase–polymerase chain reaction revealed a lower mRNA level of BCRP1 in GLC4-MITO compared with GLC4 cell line(see Figure 2

). However, the ABCA2 transporter showed a stronger signal in GLC4-MITO as compared to GLC4 indicating an increased expression level. Densitometric scanning of the PCR reaction product bands revealed a two-fold higher signal of GLC4-MITO (GLC4-MITO/HPRT: (*n*=3, ratio=2.84±0.17) compared to the GLC4 (GLC4/HPRT: (*n*=3, ratio=1.44±0.04) signal using the housekeeping gene HPRT as a loading control (triplicate experiments).

**Figure 3:**
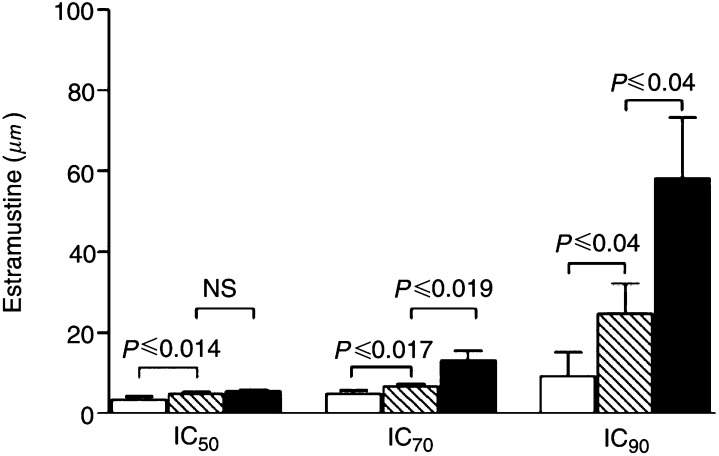
Representative cytotoxity profile of the estramustine sensitivities of GLC4-MITO (▪), GLC4-ADR (□) and GLC4 () measured with an MTA. Values represent the means of four experiments±s.d.

ABCA2 was shown by Laing *et al* (1998) to confer resistance to estramustine in an ovarian cancer cell line. In order to demonstrate functionality of ABCA2 in GLC4-MITO, a cytotoxicity assay with estramustine in GLC4-MITO, GLC4-ADR and GLC4 was performed. Figure 3

shows the result of the cytotoxity assay. A 1.5- and 2.0-fold higher drug concentration was needed to obtain the IC_70_ and IC_90_, respectively, in GLC4-MITO compared to GLC4. The GLC4-ADR cell line with increased MRP1 and LRP expression (but no reduced mitoxantrone accumulation) was more sensitive to estramustine than the GLC4 and GLC4-MITO cell line. A 2.5-fold lower drug concentration was needed to obtain the IC_90_ compared to GLC4 (see Figure 3).

**Figure 4:**
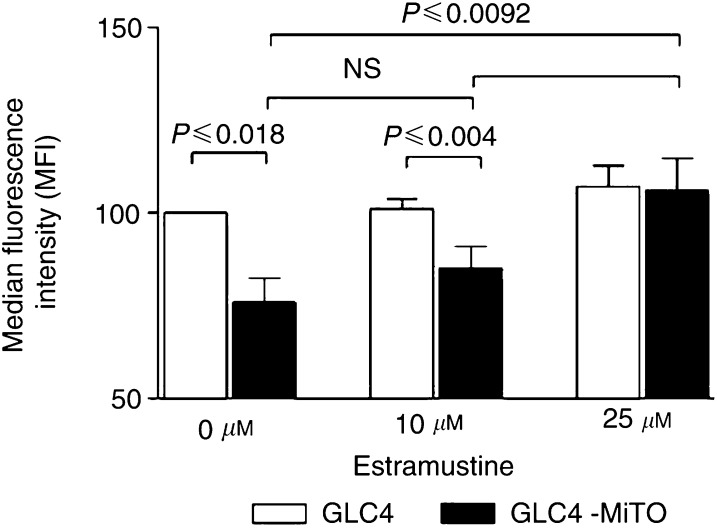
Effect of estramustine on mitoxantrone drug accumulation in GLC4 and GLC4-MITO. In total, 0, 10 and 25 *μ*M estramustine were added together with 3 *μ*M mitoxantrone to 1 × 10^6^ GLC4 and GLC4-MITO cells. Median fluorescence intensity was measured indicating the relative amount of mitoxantrone accumulation. Values represent the median mitoxantrone fluorescence relative to GLC4 with 3 *μ*M mitoxantrone. Values represent the means of three experiments±s.d. No significant difference was observed in GLC4 with or without estramustine.

The ability of tumour cell lines GLC4 and GLC4-MITO to extrude mitoxantrone, and the effect of estramustine on mitoxantrone accumulation were measured with a FACS assay. The mitoxantrone accumulation after exposure to 3 *μ*M mitoxantrone was 25% reduced in GLC4-MITO (76±11 MFI, *P*=0.03) compared to GLC4 (set as 100) (see Figure 4

). When 10 and 25 *μ*M estramustine were added during incubation with mitoxantrone, the mitoxantrone accumulation in GLC4-MITO increased to 85±10 MFI (*n*=3, *P*=0.004) with 10 *μ*M estramustine and to 106±15 MFI (*n*=3, *P*=0.03) with 25 *μ*M estramustine. However, mitoxantrone accumulation was not effected in GLC4, 101±5 MFI (*n*=3, *P*=0.41) and 107±11 MFI (*n*=3, *P*=0.16) with 10 or 25 *μ*M estramustine (see Figure 4). When 25 *μ*M estramustine was added to GLC4-MITO, the cellular mitoxantrone level increased to the same level as for GLC4 (see Figure 4). The BCRP1 (FTC), P-gp (PSC833) and MRPs (MK-571) inhibitors were added to GLC4-MITO and GLC4 prior to addition of 3 *μ*M mitoxantrone, and the drug accumulation was measured by FACS analysis. The mitoxantrone accumulation expressed as MFI was set to 100 with addition of only 3 *μ*M mitoxantrone. The addition of the P-gp inhibitor PSC833 caused an increase in mitoxantrone accumulation to 113±19 (*n*=3, *P*=0.17) in GLC4 and an increase to 114±20 (*n*=3 *P*=0.19) in GLC4-MITO. When the inhibitor for BCRP1, FTC was added, a mitoxantrone increase was detected in GLC4 (114±10 (*n*=3 *P*=0.07)) and GLC4-MITO (128±8(*n*=3, *P*=0.02)). However, there was no significant difference (*P*=0.13) in mitoxantrone accumulation between the cell lines. The addition of the MRP inhibitor MK-571 caused an increase in mitoxantrone accumulation to 117±27 in GLC4 (*n*=3, *P*=0.09) and to 119±28 (*n*=3, *P*=0.22) in GLC4-MITO.

## DISCUSSION

This study shows that with CGH it was possible to define an amplified chromosomal area, in which after a database search ABCA2 could be identified as a candidate gene involved in mitoxantrone resistance. In the mitoxantrone-resistant GLC4-MITO, mechanisms underlying the mitoxantrone accumulation defect were unknown (Withoff *et al*, 1996). Direct comparison with CGH of GLC4-MITO and its parental cell line GLC4 resulted in a simplified overview of numerical differences (see Table 2). These differences fitted in the complex karytotype obtained with cytogenetics. Although in both lines marker chromosomes were found, which made it difficult to identify chromosomal gains.

Mitoxantrone resistance in GLC4-MITO is at least partly due to the absence or low-level expression of topoisomerase II*α* and *β* compared with its parental line (Withoff *et al*, 1996). The loss of 3p (containing the topoisomerase II*β* gene) and 17q (containing the topoisomerase II*α* gene) in GLC4-MITO detected with CGH is in accordance with this previously found reduction of topoisomerase activity (Withoff *et al*, 1996).

GLC4-MITO has a low level of doxorubicin crossresistance which might be completely due to lowered topoisomerase II levels (Withoff *et al*, 1996). This implies that an uncharacterised transporter protein capable of reducing mitoxantrone accumulation without a role in doxorubicin resistance is involved in mitoxantrone resistance in GLC4-MITO.

Mitoxantrone resistance has been contributed to overexpression of various ABC transporters. The BCRP1 half-transporter protein was found to be responsible for mitoxantrone resistance (Doyle *et al*, 1998; Miyake *et al*, 1999; Ross *et al*, 1999; Litman *et al*, 2000). Scheffer *et al* (2000) had already shown that there was no BCRP1 overexpression in GLC4-MITO using BXP-34 antibody. Comparative genomic hybridisation analysis in the present study revealed the loss of the 4q13–q28 region harbouring BCRP1 (4q21–22, Knutsen *et al*, 2000) in GLC4-MITO and a lower BCRP1 mRNA expression compared with GLC4 (Figure 2A). Thus, BCRP1 is not involved in mitoxantrone resistance in GLC4-MITO. ABCB2 and ABCB3 have been associated with mitoxantrone resistance in a gastric carcinoma cell line (Lage *et al*, 2001). Mitoxantrone resistance due to a reduced expression of topoisomerase II and an unknown efflux pump was observed in human (Hazlehurst *et al*, 1999; Diah *et al*, 2001) and mouse (Nielsen *et al*, 2000) cell lines. In the present study, several genomic regions that harbour candidate drug resistance genes in the GLC4-MITO were located. In these regions, seven transporter genes were identified (Müller M (2001) *Transporters in the Liver and ATPbinding Casette (ABC)-Proteins October 2002*. *Internet communication*, http://nutrigene.4t.com/humana bc.htm). Reverse transcriptase–polymerase chain reaction for four transporter genes, ABCB6 (Allikmets *et al*, 1996), ABCF1 (Richard *et al*, 1998), ABCC4 (Kool *et al*, 1997) and ABCC10 (Hopper *et al*, 2001), and immunohistochemistry of two genes, ABCB2 and ABCB3 (Bahram *et al*, 1991), showed similar levels of mRNA and protein, respectively, in GLC4-MITO and GLC4.

However, the ABCA2 (Vulevic *et al*, 2001) gene located on 9q34 showed an increased mRNA expression in GLC4-MITO compared with GLC4. No causal link between ABCA2 and mitoxantrone resistance has been demonstrated. Gain of 9q34, including the ABCA2 transporter gene and overexpression of ABCA2 mRNA in an ovarian carcinoma cell line has been associated with enhanced efflux of estramustine (Laing *et al*, 1998). Transfection of ABCA2 in HEK293 cells resulted in a two-fold resistance to estramustine compared to normal HEK293 cells (Vulevic *et al*, 2001). ABCA2 has been suggested to play a role in the transport of steroids, lipids and related molecules (Vulevic *et al*, 2001; Zhou *et al*, 2001) and is expressed at high level in brain and neural tissue (The cancer genome anatomy project (2001): *Sage Genie, Sage Anatomic Viewer, Digital Northern for ABCA2. Internet communication*, http://cgap.nci.nih.gov/SAGE/A natomicViewer). ABCA2 expression has been observed in tumour cell lines of different origin and intracellular localisation to the endosome/lysosome compartment was demonstrated (Vulevic *et al*, 2001). Lysosomes were capable of exocytosis in response to extracellular stimuli also in nonsecretory cells (Jaiswal *et al*, 2002). In the GLC4 and the GLC4-ADR cell lines, the transport of doxorubicin by secretory vesiscles with MRP1containing membranes was previously demonstrated (van Luyn *et al*, 1998). In GLC4-MITO (and GLC4), mitoxantrone extrusion could take place analogously by the lysosome exocytosis pathway.

Analysis of the cytotoxicity of estramustine on GLC4, GLC4-MITO and GLC4-ADR showed a two-fold resistance in GLC4-MITO (see Figure 3) comparable with the estramustine resistance found in an ABCA2-transfected cell line (Vulevic *et al*, 2001). Remarkably, the multidrug-resistant, MRP1-overexpressing GLC4-ADR (Versantvoort *et al*, 1995; Withoff *et al*, 1996) was more sensitive to estramustine than the GLC4 control cell line (see Figure 3). In GLC4-MITO, addition of 25 *μ*M estramustine increased mitoxantrone accumulation to the level of GLC4 (see Figure 4), suggesting that estramustine is able to block mitoxantrone efflux in GLC4-MITO. This effect of estramustine is unlikely to be due to P-gp (Speicher *et al*, 1994; Smith *et al*, 1995) or MRP5 (Wielinga *et al*, 2003) of which estramustine has been shown to modulate the transport activity. In the present model, no P-gp overexpression and no (selective) effect of the P-gp blocker PSC833 on mitoxantrone accumulation were found. In GLC4-MITO, no increased mRNA expression was found for MRP5 or one of the other multidrug-resistance related transporters tested. Mitoxantrone accumulation with specific inhibitors of P-gp, MRPs and BCRP1 revealed an a-selective and (except for FTC) not significant increase in mitoxantrone accumulation in GLC4 as well as in GLC4-MITO.

Our data provide genetic and biochemical support for the concept that ABCA2 expression is a plausible contributory factor to the multifactorial mitoxantrone-resistant phenotype, while for other transporters no apparent changes in the expression could be correlated with mitoxantrone accumulation or cytotoxicity.
